# Physical fitness in young top level table tennis players: differences between sex, age and playing style

**DOI:** 10.3389/fspor.2023.1308960

**Published:** 2023-11-28

**Authors:** Francisco Pradas de la Fuente, Víctor Toro-Román, Miguel Ángel Ortega-Zayas, Alejandro Moreno-Azze

**Affiliations:** ^1^ ENFYRED Research Group, University of Zaragoza, Huesca, Spain; ^2^ Faculty of Sports Science, University of Extremadura, University Avenue, Cáceres, Spain

**Keywords:** racket sports, youth, elite, defensive, offensive

## Abstract

Understanding the physical fitness of table tennis (TT) players could be interesting in improving the training process and evaluating talent. This study aimed to assess the physical fitness of U14 TT players and differentiate between sex, age and playing style. A total of 352 players (203 males and 149 females) aged between 9 and 13 years participated in the present study. Furthermore, the sample was divided according to playing style: offensive (OFF) and mixed + defensive (M + D). A battery of tests was carried out to assess cardiorespiratory capacity, speed, strength, flexibility and power. Both sexes reported significant differences in cardiorespiratory capacity and speed (*p* < 0.05). Concerning age, there were substantial differences in cardiorespiratory capacity, speed, strength and power, with older players obtaining better results (*p* < 0.05). Finally, concerning playing style, differences were reported in cardiorespiratory capacity, higher in OFF style group, and flexibility, higher in the M + D style group. Finally, there were relationships between playing style and cardiorespiratory fitness and flexibility. Physical fitness evolves with increasing age as a function of sex. This is the first study to assess fitness in a large sample of TT players as a function of playing style.

## 1. Introduction

Table tennis (TT) is a sport where highly complex motor tasks are performed. The efforts made during a game in TT are acyclic, intermittent, of short duration, high intensity, with constant changes of rhythm and direction, where players must continuously react, move and hit a ball that moves at high speed (1, 2). The activity of a TT player is not only determined by the complexity of its technical and tactical actions but also by the cumulative-explosive nature of the physical effort caused by the high precision required by the movements to be executed (2) and the continuous repetitiveness of interactions between the neuromuscular system and the ball as a moving stimulus (3).

At the technical level, the dynamics of TT playing demands are characterized by the continuous and fast execution of different types of strokes and displacements that involve significant physical stress for players. Movements at high velocity and endurance are two critical physical qualities in this sport (4, 5). Short and intense efforts during the game require essential training in reaction speed and anaerobic endurance (5, 6), At the same time, the duration of the matches makes it necessary to maintain an adequate aerobic endurance base (7, 8).

The resistance to be overcome by the upper extremity when impacting on the ball is relatively small (6). However, this type of action involves a significant muscular effort for the lower extremities due to the degree of acceleration and deceleration required to hit each ball in a properly position (9, 10). In this sense, the manifestations of the lower extremities' explosive and reactive strength are considered one of the sport's main physical qualities (11, 12). On the other hand, flexibility in TT performance is of little relevance. However, the range of motion of the lower body is an essential physical aspect because if it is insufficient, there is a reduction of the gestural content and a deterioration of neuromuscular coordination (13).

In terms of tactical skills, TT is a demanding sport as athletes have to plan different and precise technical-tactical actions (14), depending on the style of play and the opponent, making decisions in very short periods of time, also enduring situations of physical fatigue (15). TT players mainly use two styles of play: offensive (OFF) and defensive (DEF) (16). Although less known, there is also a style of play called mixed (MIX), intermediate between the two previous styles. The development of each type of play is directly related to the use of specific materials in the table tennis blades (17).

The OFF style of play is one in which the players are positioned at a very close or far distance from the table, using techniques to initiate the attack, either through lift-type strokes such as flip and topspin or with blocks to accelerate the game's pace (1, 18). On the other hand, the DEF playing style is performed at a medium distance or even far away from the table. In a defensive game, the player tries to nullify the opponent's attacking strokes by defensive techniques (push or chop), producing a recoil effect on the ball. These players tend to slow down the game's speed, lengthening the play and trying to win the point by forcing their opponent to make mistakes (1, 18). Finally, the MIX playing style can be performed very close to the table or at a middle distance. This style of play is very varied and aims to confuse the opponent with different types of slice or lift effects, especially in a defensive phase (1, 18).

In the scientific literature, some studies show differences in the physical load depending on the style of play (19). Differences have been reported in physiological parameters (heart rate and maximum oxygen consumption), the duration of games and matches, and the type and number of total strokes performed (7, 19, 20). However, no research has been found that analyzes and compares the levels of physical fitness as a function of the style of play developed (OFF vs. MIX + DEF), especially in young top level players, and in particular studies that include the female sex. Consequently, the study aimed to analyze the influence of playing styles (OFF vs. MIX + DEF) in young high-level TT players' physical fitness levels according to age and sex.

## 2. Materials and methods

### 2.1. Participants

Three hundred fifty-two children aged from 9 to 13 years volunteered to participate in the study (203 males and 149 females). The sample size is larger for a population of 1981 players of U14 categories (based on the number of licenses of the Royal Spanish Table Tennis Federation) for a confidence interval of 95% and a margin of error of 5% (*n* = 322). The TT players were recruited from the National Sports Technification Program developed by the Royal Spanish Table Tennis Federation. All players were ranked in the top positions of their respective categories and sex. The playing style data were obtained by reviewing the types of racquets and coverings used by the players, in addition to observing the use given by the players during training and competitions. Also, the players and their coaches were asked about the type of game they intended to develop with the playing equipment used.

After an extensive verbal and written explanation of the study, written informed consent was obtained from the parents or legal guardians. The research protocol was reviewed and approved by the Clinical Research Ethics Committee of Aragon (Spain) (code: 19/2010) following the guidelines of the Helsinki Ethical Declaration for research in humans.

For inclusion in the study, participants had to achieve the following criteria: (i) practice only TT as a sport modality; (ii) have at least one year's experience; (iii) be federated; (iv) not having any pathology; (v) not ingest of medication. The playing characteristics of the participants were provided by the coaches of the study participants (Table 1).

**Table 1:** General characteristics of the sample.

Variables		*n*	%
Sex	Male	203	57.6
	Female	149	42.4
Playing style	Defensive	5	1.4
	Mixed	12	3.4
	Offensive	335	95.2
Laterality	Right-handed	318	90.3
	Left-handed	34	9.7
	9	32	9.1
	10	80	22.7
	11	94	26.7
	12	109	31.0
	13	37	10.5

### 2.2. Procedures

The study was carried out during the summer months, coinciding with the vacation period of the participants. No training was required for a more objective assessment the day before the evaluations.

Before the assessments, participants performed a 10-minute warm-up based on general mobility and jogging. All participants were familiarized with the different tests. The physical fitness assessments were performed in two different phases in the following order: anthropometry, flexion, vertical jump, upper limb strength and running speed. In the second phase, carried out 60 min after the first phase, the test to assess maximum cardiorespiratory capacity was performed.

### 2.3. Anthropometric

Body weight, height and body mass index (BMI) were evaluated. A scale (Seca 769, Seca, Hamburg, Germany) and a measuring rod (Seca 220, Seca, Hamburg, Germany) were used. All participants were evaluated with as minimal clothing as possible and without footwear.

### 2.4. Flexibility

The sit-and-reach test was used to measure the range of motion of the lumbar region and the hamstring muscle, according to standardized procedures (21) using the sit-and-reach flexibility tester (Baseline®, New York, USA). From a seated position on the floor with legs fully extended, participants extended the arm along the measurement scale as far as possible without bending the knee, placing one hand on the other with palms down. The best of two repetitions was chosen for further analysis.

### 2.5. Vertical jump

To evaluate the vertical jump, the squat jump (SJ), counter-movement jump (CMJ) and Abalakov jump (ABK) tests were established (22). A jump mat system (Newtest Powertimer®, Oulu, Finland) was used to measure height and flight time during the jumps. During the test, the guidelines proposed by Bosco et al. (23), were followed.

For the SJ, participants initiated the movement from a squat position (knee angle 90°) and arms resting on the hips. After 2 s of holding the position, a jump was performed without countermovement at maximum intensity. For the CMJ, participants started the execution from an upright position and hands resting on the hips. Subjects performed a knee flexion-extension followed by a jump at maximum possible intensity. For the ABK, participants could propel their arms by swinging.

Recovery was 30 s between jumps. The best jump of three attempts was selected for further analysis.

### 2.6. Upper limb strength

The handgrip strength and medicine ball throw tests were performed to assess upper body strength.

A Takei 5,101 dynamometer (Takei Instruments Ltd., Tokyo, Japan) was used to determine handgrip strength. Participants completed two maximal voluntary contractions with the dominant and non-dominant hand. At all times, the arm was extended. The grip of the dynamometer was adjusted to the participant's hands. The best of two alternative repetitions was chosen. The asymmetry of grip strength (difference between the dominant and non-dominant hand) was evaluated.

The overhead medicine ball throw tests upper body strength and explosive power, which consists of throwing the ball forward over the head. It consists of performing a quick downward motion, bending the knees and hips, before quickly extending the hips, knees and arms to throw the medicine ball as far as possible. A 3 kg medicine ball was used for both sexes. Two attempts were made, and the most significant distance obtained was chosen for analysis.

### 2.7. Flying 30 meter sprint test

Flying 30-meter sprint test is a physical test used to measure the maximum sprint speed of an individual. On a 70-meter straight line, the subject sprints to reach the maximum speed upon reaching a photocell located at 20 m and holding it until passing the photocell at the finish line located at 50 m (Newtest Powertimer®, Oulu, Finland). Two attempts were performed with a 2-minute rest between attempts. The best of the two repetitions was chosen.

### 2.8. Cardiovascular fitness

Cardiovascular fitness was examined by a maximal multistage 20 m shuttle run test (Course Navette Test; CN) (24). Sound signals were emitted from a pre-recorded tape that increased 0.5 km·h^−1^ each minute from a starting speed of 8.5 km·h^−1^. When the subject could no longer follow the pace, the last stage number announced was used to estimate the maximal oxygen uptake (VO_2max_) by formula (24). Distance, periods, and speed were noted.

### 2.9. Statistical analysis

The data were processed in IBM SPSS 25.0 Statistics (IBM Corp., Armonk, NY, USA) and were expressed as mean ± standard deviation, except for the data on the characteristics of the participants, which were described as frequencies and percentages. The normality of the distribution of the variables was analyzed using the Kolmogorov-Smirnov test and the homogeneity of variances using the Levene test. A two-way ANOVA (sex and age effect) was used to show any differences in the variables studied. The effect size was calculated using partial eta squared. Effect size values were classified as 0.01–0.06 small effect size; 0.06–0.14 moderate effect size; >0.14 large effect size (25). A *t*-test for independent samples was used to analyze differences between play styles. Finally, Pearson's correlation coefficient *r* was used to establish relationships between playing style and sex with the physical fitness parameters analyzed. Differences of *p* < 0.05 were considered statistically significant.

## 3. Results

The data obtained in the present study are presented below. Table 2 shows the anthropometric characteristics. Significant differences were observed in height, weight and BMI (*p* < 0.05) being higher in older players.

**Table 2:** Anthropometric characteristics.

Variables	Age (years)	Male	Female	Sex effect	Age effect	Sex × Age
Height (m)	9	1.39 ± 0.05	1.38 ± 0.06	0.301	<0.001^##^	0.510
	10	1.42 ± 0.07	1.43 ± 0.05			
	11^^^^	1.47 ± 0.06	1.50 ± 0.07			
	12^^^,$$^	1.52 ± 0.06	1.54 ± 0.06			
	13^^^^^,^^$$^^,^^&&^	1.57 ± 0.08	1.56 ± 0.06			
Weight (kg)	9	33.61 ± 4.15	34.02 ± 7.87	0.443	<0.001^##^	0.496
	10	37.02 ± 7.10	38.15 ± 6.28			
	11^^^^	41.08 ± 9.04	42.92 ± 8.93			
	12^^^^	44.28 ± 7.72	44.59 ± 6.35			
	13^^^,$$,&&^	50.72 ± 9.71	47.00 ± 6.89			
BMI (kg/m^2^)	9	17.26 ± 1.85	17.73 ± 3.68	0.515	0.019^##^	0.510
	10	18.22 ± 2.59	18.57 ± 2.83			
	11	18.68 ± 3.20	18.81 ± 3.15			
	12	19.04 ± 2.50	18.62 ± 1.94			
	13	20.34 ± 2.79	19.13 ± 2.28			

^^ *p* < 0.01 differences vs. 9.

^$$^ *p* < 0.01 differences vs. 10.

^&&^ *p* < 0.01 differences vs. 11.

^##^ Large effect size.

The results related to the upper body strength and flexibility are presented in Table 3. There were significant differences between sexes in medicine ball throwing and flexibility (*p* < 0.001). On the other hand, with respect to age, there were differences in hand grip and medicine ball throwing, being higher in older players (*p* < 0.001).

**Table 3:** Upper body strength and lower body posterior flexibility.

Variables	Age (years)	Male	Female	Sex effect	Age effect	Sex × Age
HG Dominant (kg)	9	17.78 ± 2.69	15.01 ± 2.27	0.490	<0.001^##^	0.018^##^
	10	17.03 ± 3.68	17.14 ± 3.18			
	11^^^,$$^	20.27 ± 4.32	18.04 ± 2.38			
	12	23.84 ± 5.21	23.81 ± 3.65			
	13	25.48 ± 6.76	24.78 ± 5.13			
HG Non-Dominant (kg)	9	16.88 ± 2.51	13.84 ± 2.24	0.181^#^	<0.001^##^	0.014^##^
	10	15.10 ± 3.14	15.54 ± 3.30			
	11^^^,$$^	17.70 ± 4.48	16.32 ± 2.60			
	12	21.14 ± 5.46	21.84 ± 5.13			
	13	24.44 ± 5.43	22.10 ± 3.17			
Asymmetry HG (%)	9	3.47 ± 11.71	12.29 ± 4.90			
	10	9.23 ± 8.62	15.54 ± 9.75	0.312	0.542	0.601
	11	8.62 ± 9.38	14.14 ± 4.87			
	12	9.61 ± 10.65	12.71 ± 9.36			
	13	10.61 ± 12.47	12.78 ± 10.20			
Medicine ball throw (m)	9	3.01 ± 0.39	2.18 ± 0.18	<0.001^##^	<0.001^##^	0.037^##^
	10	3.20 ± 0.41	2.62 ± 0.52			
	11^^^^	3.64 ± 0.44	3.12 ± 0.46			
	12^^^,$$,&&^	4.33 ± 0.84	3.66 ± 0.74			
	13^^^,$$,&&,^“”	5.45 ± 1.00	4.42 ± 0.37			
Flexibility (cm)	9	16.28 ± 4.19	21.33 ± 8.03	<0.001^##^	0.121^#^	0.043^##^
	10	14.18 ± 3.21	21.50 ± 6.27			
	11	17.10 ± 5.09	23.50 ± 6.03			
	12	18.84 ± 5.16	23.71 ± 8.44			
	13	19.67 ± 4.16	20.16 ± 6.43			

HG, hand grip.

^^ *p* < 0.01 differences vs. 9.

^$^ *p* < 0.05 differences vs. 10.

^&^ *p* < 0.05 differences vs. 11.

\" *p* < 0.01 differences vs. 12.

^##^ Large effect size.

^#^ Moderate effect size.

The results related to the vertical jump are presented in Table 4. No significant differences were observed between sexes. However, there were significant differences between the ages of the participants where the jump height was higher with increasing age (*p* < 0.001).

**Table 4:** Vertical jump.

Variables	Age (years)	Male	Female	Sex effect	Age effect	Sex × Age
SJ (cm)	9	17.67 ± 2.41	17.55 ± 3.21	0.434	<0.001^##^	0.911
	10	19.02 ± 3.91	18.53 ± 4.43			
	11	21.01 ± 4.37	20.15 ± 4.01			
	12^^^,$$^	22.81 ± 4.67	22.52 ± 4.26			
	13^^^,$$^	24.18 ± 4.99	22.59 ± 3.48			
CMJ (cm)	9	20.80 ± 2.76	21.66 ± 4.17	0.382	<0.001^##^	0.932
	10	21.64 ± 4.02	20.87 ± 5.09			
	11	24.29 ± 4.59	23.70 ± 4.20			
	12^^^,$$^	26.07 ± 5.64	25.40 ± 5.04			
	13^^^$$^	27.60 ± 5.38	25.55 ± 3.02			
ABK (cm)	9	23.58 ± 3.69	23.13 ± 4.11	0.670	<0.001^##^	0.714
	10	23.80 ± 4.88	23.82 ± 6.58			
	11	27.48 ± 5.09	27.58 ± 4.72			
	12^^^,$$^	29.79 ± 6.00	28.81 ± 6.07			
	13^^^,$$^	31.66 ± 5.5	29.50 ± 4.75			

SJ, squat jump; CMJ, countermovement jump; ABK, abalakov jump.

^^^^ *p* < 0.01 differences vs. 9.

^$$^ *p* < 0.05 differences vs. 10.

^##^ Large effect size.

Table 5 shows the results obtained in velocity and cardiorespiratory test. There were significant differences and large effect sizes between sexes and between ages in all parameters analyzed (*p* < 0.05).

**Table 5:** Cardiorespiratory capacity and speed assessment.

Variables	Age (years)	Male	Female	Sex effect	Age effect	Sex × Age
Periods CN (n)	9	4.21 ± 1.40	3.28 ± 0.84	<0.001^##^	<0.001^##^	0.516
	10	4.93 ± 1.56	3.53 ± 1.09			
	11^^^,$$^	6.35 ± 1.58	4.37 ± 1.25			
	12^^^,$$^	6.19 ± 1.53	4.89 ± 1.56			
	13^^^^	7.00 ± 1.98	5.30 ± 1.29			
Speed CN (km/h)	9	10.12 ± 0.69	9.67 ± 0.42	<0.001^##^	<0.001^##^	0.421
	10	10.55 ± 0.79	9.85 ± 0.55			
	11^^^^	11.24 ± 0.81	10.26 ± 0.62			
	12^^^,$$^	11.15 ± 0.77	10.51 ± 0.80			
	13^^^^	11.55 ± 1.02	10.76 ± 0.66			
Distance CN (m)	9	658.4 ± 241.8	502.5 ± 139.6	<0.001^##^	<0.001^##^	0.561
	10	812.7 ± 291.1	564.4 ± 191.3			
	11^^^^	1,068.3 ± 311.6	706.0 ± 221.6			
	12^^^,$$^	1,033.4 ± 297.1	798.1 ± 288.8			
	13^^^^	1,178.3 ± 379.5	887.3 ± 246.1			
VO_2max_ (mL/min/kg)	9	39.85 ± 4.07	37.23 ± 2.46	<0.001^##^	<0.001^##^	0.481
	10	42.36 ± 4.67	38.28 ± 3.27			
	11^^^^	46.38 ± 4.78	40.67 ± 3.64			
	12^^^,$$^	45.86 ± 4.56	42.12 ± 4.69			
	13^^^^	48.19 ± 6.00	43.62 ± 3.89			
Speed 20 + 30 m (s)	9	5.48 ± 0.70	6.14 ± 1.07	0.019^##^	<0.001^##^	0.463
	10	5.60 ± 1.01	5.81 ± 0.99			
	11	5.03 ± 0.56	5.29 ± 0.65			
	12^^^,$$^	4.85 ± 0.39	5.03 ± 0.31			
	13^^^,$$^	4.53 ± 0.42	4.96 ± 0.39			

VO_2max_, maximal oxygen uptake; CN, course navette.

^^^^ *p* < 0.01 differences vs. 9.

^$$^ *p* < 0.01 differences vs. 10.

^&&^ *p* < 0.01 differences vs. 11.

^##^ Large effect size.

Table 6 shows the differences in the physical fitness parameters analyzed previously comparing between playing styles. Differences between playing styles were observed in weight (*p* = 0.012), CN period (*p* = 0.017), CN distance (*p* = 0.033), CN speed (*p* = 0.033), maximum oxygen consumption (*p* = 0.033) and flexibility (*p* < 0.001).

**Table 6:** Differences in physical fitness in all participants according to playing style.

Variables	Ofensive	Mixed-Defensive	*t*	*p*
Height (m)	1.47 ± 0.070	1.51 ± 0.07	1.7	0.09
Weight (kg)	41.05 ± 7.11	46.71 ± 6.08	2.5	0.01
BMI (kg/m^2^)	18.71 ± 2.19	20.21 ± 1.34	2.3	0.01
Periods CN (n)	5.24 ± 1.73	4.11 ± 1.49	2.3	0.01
Distance CN (m)	861.02 ± 320.51	677.47 ± 270.45	2.1	0.03
Speed CN (km/h)	10.86 ± 0.93	10.17 ± 0.74	2.1	0.03
VO_2max_ (mL/min/kg)	44.1 ± 5.21	40.15 ± 4.38	2.1	0.03
Speed 20 + 30 (s)	5.28 ± 0.71	5.19 ± 0.67	0.2	0.81
HG Dominant (kg)	21.16 ± 5.41	24.08 ± 7.05	1.9	0.05
HG Non-Dominant (kg)	19.07 ± 4.15	20.63 ± 6.12	1.3	0.17
Asymmetry HG (%)	10.87 ± 8.10	13.97 ± 9.67	1.0	0.29
Medicine ball throw (m)	3.85 ± 0.83	3.78 ± 0.97	0.3	0.70
Flexibility (cm)	18.43 ± 4.42	24.20 ± 9.73	3.8	<0.01
SJ (cm)	21.06 ± 4.59	20.87 ± 5.15	0.0	0.99
CMJ (cm)	24.21 ± 6.06	24.17 ± 5.44	0.2	0.84
ABK (cm)	28.01 ± 5.13	26.32 ± 6.83	0.5	0.60

BMI, body mass index; CN, course navette; VO_2max_, maximal oxygen uptake; HD, hand grip; SJ, squat jump; CMJ, countermovement jump; ABK, abalakov jump.

Table 7 shows the differences in physical fitness parameters as a function of playing style and sex. Regarding playing style, significant differences were observed in BMI (*p* = 0.025). In relation to sex, no significant differences were reported.

**Table 7:** Anthropometry and physical fitness according to sex and playing style**.**

	Style	Male	Female	Sex effect	Style effect	Style × Age
Height (m)	OFF	1.46 ± 0.05	1.45 ± 0.04	0.812	0.710	0.910
	MIX + DEF	1.48 ± 0.02	1.47 ± 0.11			
Weight (kg)	OFF	41.14 ± 8.81	38.61 ± 7.34	0.317	0.081^##^	0.615
	MIX + DEF	49.10 ± 2.12	44.38 ± 7.98			
BMI (kg/m^2^)	OFF	19.10 ± 4.12	18.51 ± 3.09	0.276	0.022^##^	0.498
	MIX + DEF	22.26 ± 0.32	20.19 ± 0.82			
Periods CN (n)	OFF	5.64 ± 1.61	4.21 ± 1.19	0.691	0.114^#^	0.101^#^
	MIX + DEF	3.50 ± 0.00	4.30 ± 1.30			
Distance CN (m)	OFF	958.5 ± 351.9	710.5 ± 231.8	0.495	0.131^#^	0.211
	MIX + DEF	608.0 ± 0.0	681.2 ± 199.9			
Speed CN (km/h)	OFF	11.09 ± 0.61	10.51 ± 0.61	0.412	0.181^#^	0.191
	MIX + DEF	10.0 ± 0.0	10.20 ± 0.57			
VO_2max_ (ml/min/kg)	OFF	45.12 ± 4.18	41.01 ± 3.91	0.517	0.161^#^	0.191
	MIX + DEF	39.12 ± 0.00	40.29 ± 3.33			
Speed 20 + 30 (s)	OFF	5.21 ± 0.79	5.45 ± 0.61	0.415	0.463	0.911
	MIX + DEF	5.43 ± 0.19	5.63 ± 0.85			
HG Dominant (kg)	OFF	19.41 ± 4.91	23.36 ± 4.92	0.101^#^	0.681	0.517
	MIX + DEF	20.35 ± 7.42	23.14 ± 6.57			
HG Non Dominant (kg)	OFF	17.42 ± 4.76	19.99 ± 5.42	0.253	0.955	0.966
	MIX + DEF	17.40 ± 5.09	19.78 ± 6.74			
Asymmetry HG (%)	OFF	8.19 ± 9.11	12.17 ± 7.55	0.321	0.411	0.691
	MIX + DEF	13.29 ± 6.61	14.94 ± 11.53			
Medicine ball throw (m)	OFF	3.91 ± 0.91	3.41 ± 0.95	0.085^##^	0.714	0.615
	MIX + DEF	4.03 ± 0.80	3.26 ± 0.87			
Flexibility (cm)	OFF	17.15 ± 3.11	20.15 ± 7.18	0.055^##^	0.796	0.618
	MIX + DEF	16.00 ± 1.41	21.50 ± 10.01			
SJ (cm)	OFF	22.18 ± 5.81	20.81 ± 4.27	0.312	0.219	0.610
	MIX + DEF	19.50 ± 0.70	16.76 ± 2.56			
CMJ (cm)	OFF	24.69 ± 6.94	22.91 ± 6.11	0.415	0.131	0.619
	MIX + DEF	21.85 ± 0.49	19.88 ± 3.47			
ABK (cm)	OFF	28.12 ± 7.49	27.39 ± 6.12	0.421	0.181	0.487
	MIX + DEF	25.50 ± 0.70	21.60 ± 4.27			

BMI, body mass index; CN, course navette; VO_2max_, maximal oxygen uptake; HD, hand grip; SJ, squat jump; CMJ, countermovement jump; ABK, abalakov jump; IE, elastic index.

^##^ Large effect size.

^#^ Moderate effect size.

Finally, Table 8 shows the correlations between physical condition parameters and playing style as a function of sex. There were negative correlations in male players and the CN period (*p* = 0.045). That is, OFF players performed longer periods in the CN. Regarding female players, there were positive correlations between female players and maximal oxygen consumption (*p* = 0.037), as well as flexibility (*p* = 0.030). That is, players with MIX and DEF playing styles obtained higher values of maximal oxygen consumption and flexibility.

**Table 8:** Correlations, according to sex, in the parameters of physical fitness and style of play.

			Playing style (0 = OFF; 1 = MIX + DEF)
Periods CN (n)	Male	*r*	−0.144
		*p*	0.043
	Female	*r*	−0.004
		*p*	0.961
Distance CN (m)	Male	*r*	−0.110
		*p*	0.117
	Female	*r*	0.007
		*p*	0.930
Speed CN (km/h)	Male	*r*	−0.110
		*p*	0.111
	Female	*r*	0.006
		*p*	0.941
VO_2max_ (mL/min/kg)	Male	*r*	−0.105
		*p*	0.131
	Female	*r*	0.005
		*p*	0.961
Speed 20 + 30 (s)	Male	*r*	0.042
		*p*	0.541
	Female	*r*	−0.101
		*p*	0.225
HG Dominant (kg)	Male	*r*	0.041
		*p*	0.681
	Female	*r*	0.115
		*p*	0.351
HG Non-Dominant (kg)	Male	*r*	0.004
		*p*	0.961
	Female	*r*	0.099
		*p*	0.411
Medicine ball throw (m)	Male	*r*	0.010
		*p*	0.891
	Female	*r*	0.712
		*p*	0.128
Flexibility (cm)	Male	*r*	−0.004
		*p*	0.951
	Female	*r*	0.171
		*p*	0.034
SJ (cm)	Male	*r*	−0.031
		*p*	0.631
	Female	*r*	0.041
		*p*	0.591
CMJ (cm)	Male	*r*	−0.043
		*p*	0.551
	Female	*r*	0.072
		*p*	0.381
ABK (cm)	Male	*r*	−0.034
		*p*	0.612
	Female	*r*	−0.015
		*p*	0.836

BMI, body mass index; CN, course navette; VO_2max_, maximal oxygen uptake; HD, hand grip; SJ, squat jump; CMJ, countermovement jump; ABK, abalakov jump.

## 4. Discussion

The objective of the present study was to analyze the differences of sex, age and style of play on the physical fitness of young high-level TT players. The present study reported significant differences between sexes and ages in the physical fitness parameters. In addition, as a novelty of the current investigation, it is shown that there could also be differences in the physical fitness parameters according to the style of play, as well as correlations. However, it should be noted that the participants in each playing style group (OFF and MIX + DEF) were very heterogeneous. The different playing styles result in specific and different spatiotemporal activity patterns in terms of stroke length, stroke speed, tactics, techniques and effects on the ball, as well as using other specific materials on the table tennis blades (rubbers and wood), factors that alter the pattern of playing activity (26). Consequently, the development of one or the other style of play is related to specific physical and physiological demands associated with the performance of more explosive game actions as in the OFF style (27).

The results of the sex differences observed in the current study are similar to those reported by other authors (28–30), who reported that men have higher performance in strength, vertical jump, running speed and aerobic endurance tests. In comparison, women are significantly more flexible than men (31).

The evaluation of physical fitness using test batteries allows monitoring the evolution of the athlete to create individual training programs (6). In TT, high speed, agility, coordination, reaction time, strength and flexibility are fundamental to performing the different techniques and tactics correctly (32, 33). Previous studies have analyzed physical fitness in adult high-level TT players (6, 33) and in children practicing TT (34). However, this is the first study to assess fitness as a function of playing style.

Physical fitness is an essential marker of health and sports performance in youth (35). Proper physical fitness monitoring can be an excellent strategy to promote health and identify young athletic talent for all types of sports (35). It is common practice to analyze the physical profile of young athletes and compare them with adult athletes when aiming to predict potential success (36). However, this type of methodology is strongly rooted in assumptions (35). Adolescence is a dynamic period characterized by the growth and development of different organ systems (e.g., bone tissue, muscle tissue), which rarely progresses at the same time (37). The development of physical fitness in children and the effects of moderating variables, such as age and sex, are well documented (38, 39). Girls have more advanced skeletal and sexual maturity relative to chronological age, entering puberty and reaching peak growth velocity earlier than male (an average of 12 years in female and 14 years in male) (40).

The present study observed age differences in anthropometric parameters (weight, height, and BMI). Previous studies reported that the onset of accelerated height growth is around 10–12 years of age (41). The data on height and weight coincide with other studies performed on soccer (42), padel (43) and TT (33) players. These differences are directly related to sexual dimorphism between males and females (44, 45).

Regarding upper body strength, in the present study, differences between ages were observed in grip strength, being higher in older players. With respect to medicine ball throwing, males threw the ball a longer distance than females. These results coincide with general populations (46), young padel (47) and tennis (48) players. Sex differences in strength could be due to differences in muscle mass. It is well known that males tend to have more lean mass than females, influencing strength levels (49, 50). Sex differences in strength could be due to differences in muscle mass. It is well known that males tend to have more lean mass than females, influencing strength levels (39).

In relation to the flexibility of the lower back and hamstring muscles, the present study reported differences between sexes, with females obtaining higher values. These results coincide with other racket sports, such as padel and tennis (5, 51). The anatomical structure of the hip and pelvis could explain these differences (52), and the greater muscle mass that characterizes the male sex (41, 53). On the other hand, the differences found according to the style of play could be explained by the physical requirements of each style. The OFF style requires greater muscle mass to generate high levels of explosiveness that could be related to lower values in the sit and reach test. In this sense, a lower muscle mass in MIX + DEF players would directly impact higher flexibility values as this style of play requires less power than OFF.

As for the data obtained in the vertical jump, differences between ages were reported, with the height of the jumps being greater as age advanced. Pradas de la Fuente et al. (11), showed similar results in vertical jump performance, where significant differences were found in table tennis players from under 11 years to 17 years of age. Other authors reported similar results in school populations (54). The differences between ages could be related to higher plasma testosterone levels in males (55), as well as the development of muscle mass and anatomical changes in both sexes (38). On the other hand, the sex difference in jumps that involved a countermovement might indicate that males were slightly more effective in using the stretch-shortening cycle (56) and/or involving the hip extensor muscles (57). In the CMJ, men appear to apply greater concentric momentum and, therefore, achieve greater velocity during most of the concentric phase, including the take-off (28). When comparing jump height as a function of gender and playing style, a higher jump height, although not significant, is observed in players who play an OFF game. These data point to a tendency in the OFF style of play from an early age to generate higher levels of strength in the active (impulsive) and reactive (elastic-impulsive) manifestations as has been demonstrated in similar research (6, 58, 59).

Regarding the speed test, differences in displacement time between sexes and age were reported. Previous authors observed similar results in distances of 5 and 10 meters in TT players (33), as well as reaction times and lateral displacement times (6). The differences in times in the tests between sex and age could be related to hormonal characteristics. Higher levels of circulating testosterone, characteristic of men, result in increased muscle weight and more significant muscle cross-sectional area, translating into greater applications of reaction forces, which generates superior running performance for men (60). On the other hand, the differences found in speed as a function of playing style could be explained by the physical requirements of each playing style. The OFF style requires more muscle mass to generate high levels of reactivity and explosiveness as opposed to the more conservative and less powerful MIX + DEF style of play (6, 27).

Finally, concerning the cardiorespiratory capacity, differences were observed between sexes and ages, with greater distance and maximum oxygen consumption (VO_2max_), estimated in males and at older ages. These data align with those reported Pradas et al. (34), Pradas et al. (43), in TT and padel players. Generally, higher VO_2max_ values have been observed in males (61) and sex differences increase as they progress through adolescence. These could be attributed to males' greater muscle mass and hemoglobin concentration (61). In addition, it could also be explained by a slight increase in body fat in children aged 7–12 years, as well as a reduction in body fat at puberty (62). OFF styles of play, characterized by short and very explosive actions, show lower values of oxygen consumption compared to players who develop MIX or DEF styles of play, characterized by the development of game actions of longer duration and with a higher volume of hits and displacements, requiring a more significant contribution of the aerobic pathway (6, 26).

Concerning the differences found according to playing styles, one of the most exciting findings, considering the results obtained for both sexes, is that those players with a greater body weight tend to develop MIX + DEF playing styles, which require less explosiveness in their playing actions compared to OFF players, who need a more significant development of strength and speed. The MIX − DEF style of play at early ages is an essential technical-tactical resource to improve performance. In young TT players, the MIX − DEF style of play can be linked to different limitations, which can be physical because they have a greater body weight, cognitive because they have not yet consolidated the learning of such vital aspects in this sport as reading and analyzing quickly and effectively the effects produced on the ball; or of a technical-technical-tactical type, by covering one side of the racquet with a defensive covering (e.g., long pimples-out rubber), which prevents the opponent from benefiting from a weakness on one side of the game (1).

According to sex, no significant differences were found, but co-relationships were found. Male players with an OFF style of play were negatively correlated with the CN period (*p* < 0.05). These results could be explained as a consequence of the needs of the OFF style of play, characterized by the development of short, fast and explosive game actions, where the anaerobic metabolic pathway is essential to obtain optimal performance in this type of players (6, 19).

In the female sex, players who developed a MIX + DEF style of play were correlated with higher values of flexibility (*p* < 0.05). The MIX + DEF style of play is characterized by the performance of game actions of longer duration but at a slow pace. This type of game, in which low-intensity aerobic actions predominate, produces adaptations in the biotype, particularly in women with MIX − DEF playing styles, characterized by lower power and speed of play, and as a consequence, makes it possible to acquire and maintain higher levels of flexibility (6, 12, 33).

This study also has certain limitations: (i) the rubber materials used in the table tennis blade were not taken into consideration; (ii) the lateral dominance of the players was not considered; (iii) the years of experience not been considered; (iv) this study did not consider biological maturation; and (v) the play style and age groups were very heterogeneous in terms of the number of participants.

## 5. Conclusions

There are differences in physical fitness parameters between playing styles, sex and age. Generally, male and older players perform better in physical fitness tests.

Flexibility and cardiorespiratory capacity are related to playing style and sex. Players practising the MIX − DEF style of play have higher levels of flexibility, while those of the OFF style are characterized by developing a greater aerobic capacity.

The MIX − DEF style of play is developed in both sexes by players who have a greater body weight, so using this style of play could be considered as a technical-tactical resource to improve the performance of young athletes with certain deficiencies such as low physical fitness.

It is necessary to carry out new studies to confirm the results obtained in this study, especially at early ages, since, in some cases, the players have a short sporting experience in table tennis.

The existing differences in the levels of physical fitness could help coaches develop specific training programs according to the style of play of their players and sex.

## Data Availability

The raw data supporting the conclusions of this article will be made available by the authors, without undue reservation.
